# Influence of Dietary Habits on Macular Pigment in Childhood

**DOI:** 10.3390/jcm14082668

**Published:** 2025-04-14

**Authors:** Víctor Ponce-García, María-José Bautista-Llamas, Marta-C. García-Romera

**Affiliations:** Vision Research Group (CIVIUS), Department of Physics of Condensed Matter, Optics Area, University of Seville, 41012 Seville, Spain; vicpongar@alum.us.es (V.P.-G.); mbautista5@us.es (M.-J.B.-L.)

**Keywords:** Mediterranean diet, macular pigment, lutein, zeaxanthin, children, macular carotenoids

## Abstract

**Background/Objectives:** To analyze the macular pigment optical density (MPOD) values in a child population and to evaluate the relation between MPOD and adherence to the Mediterranean diet using a validated questionnaire specially created for children. Eighty-eight children were included in this cross-sectional study from two primary education schools of Seville (Spain). **Methods:** MPOD values were measured using Macular Pigment Screener II ^®^. Lutein and Zeaxanthin intake was evaluated by KIDMED questionnaire, which classifies children according to adherence to the Mediterranean diet. A whole ocular exam with slit-lamp biomicroscopy was conducted by a specialized optometrist. **Results:** The mean age ranged between 6 and 8 years. The mean MPOD value was 0.46 ± 0.18. The mean score of the KIDMED questionnaire was 7.19 ± 1.85. No statistically significant differences were found as a function of gender or among Mediterranean diet adherence groups. No significant differences in MPOD values between answers were found in any KIDMED questions. No correlations were found for MPOD with several variables, such as Mediterranean diet adherence and KIDMED score. **Conclusions:** MPOD levels in children could not be correlated with Mediterranean Diet adherence and, thus, good dietary habits. Genetic characteristics, mother’s diet habits, oxidative stress, and body fat composition in children could be the main factors influencing MPOD levels.

## 1. Introduction

Nowadays, it is well known that good dietary habits are fundamental for the correct development of human body composition, the promotion of cognitive function and visual health and protection against numerous diseases. The Mediterranean diet is considered to be one of the best diet patterns in the world, as it can prevent the risk of cardiovascular disease, cancer, diabetes or obesity due to its high content of extra virgin olive oil, leafy green vegetables, fruits, nuts, legumes and eggs, all of which are rich in vitamins, minerals and xanthophyll carotenoids [1,2,3], such as Lutein (L) and Zeaxanthin (Z), which cannot be synthetized by the human body.

Although these carotenoids are present in several human organs like adipose tissue, brain, kidney and liver, L and Z are preferentially accumulated in the macular pigment, over the macula lutea, which is the central region of the retina where the visual ability is maximal [4].

L and Z have beneficious properties, such as their antioxidant and anti-inflammatory power, in addition to their ability to absorb short wavelength blue light. Oxidative stress may accelerate the production of reactive oxygen species (ROS), leading to inflammatory signaling, cellular dysfunction and cell death. As a result, several diseases are associated with oxidative damage such as eye disorders, cardiovascular diseases, cancer and neurodegenerative disorders. Nevertheless, thanks to the antioxidant properties of lutein and zeaxanthin, higher MPOD levels can inhibit free radical formation, preventing lipid peroxidation and retinal pigment epithelium death and ultimately neutralizing ROS [5]. These properties could prevent the risk of retinal oxidative damage, which leads to age-related macular degeneration [4,6]. Moreover, a significant correlation between macular pigment optical density (MPOD), serum levels of L and Z, and dietary intake has been found in several studies [7,8], especially in serum levels of L and Z, in which a significant correlation with MPOD levels may exist owing to the ability of absorption by the mucosa of the small intestine, transport by low and high-density lipoprotein and storage in several human tissues [9]. In this way, MPOD could be considered as a biomarker of good dietary habits. In addition, several risk factors may affect MPOD levels, such as ethnicity, age, gender, body fat composition, smoking or physical exercise [6].

Therefore, it is important to raise awareness in order to consume L and Z in different ways (dietary intake or supplementation), due to the important role that MPOD levels could play in cerebral and foveal maturation [6].

Although the relationship between MPOD and L and Z intake to prevent retinal diseases is widely known in the adult population, there is a lack of scientific evidence in the child population. In fact, knowing the role of retinal carotenoids in childhood could provide knowledge about child vision protection and help to prevent oxidative retinal diseases in adulthood.

A pilot study was performed by Garcia-Romera et al. [10], in which 27 children were evaluated. Some general factors were analyzed, such as prevalence of refractive status, L and Z intake, body mass index (BMI), and Light-Emitting Diodes (LEDs) exposure. Nevertheless, that study did not categorize results by gender, and the findings required an enlarged sample size to provide greater clarity.

Therefore, the aim of this study was (1) to analyze the MPOD values in a child population by increasing the sample size and (2) to evaluate the relation between MPOD and Mediterranean diet adherence using a validated questionnaire specifically created for children: the KIDMED questionnaire [11]. Additionally, this study categorizes data by gender, addressing the lack of evidence regarding the role of MPOD in childhood.

## 2. Materials and Methods

### 2.1. Subjects

In order to enlarge the sample size used in the prior study and to reinforce the previously described hypothesis [10], eighty-eight children were recruited for this cross-sectional study, which was authorized by the Ethics Committee of the University of Seville and adhered to the tenets of the Declaration of Helsinki. The participants were students from two primary education schools of Seville (Spain). Previously, the parents of all subjects included in the current study were informed about the aim of this study and provided their written informed consent. A whole ocular exam with slit-lamp biomicroscopy was conducted by a specialized optometrist.

The inclusion criteria were children aged between 6 and 8 years and capable of performing the task with a MPOD counter. The exclusion criteria were: lack of written informed consent from the parents, the presence of any ocular or systemic disease (diabetes mellitus), any medications taken routinely, an incomplete dietary questionnaire, and being unable to perform the MPOD task due to distraction or a lack of understanding.

Although this study is a follow-up to a previous study, a complete dietary intake evaluation was performed, categorizing by gender and not analyzing BMI or LEDs exposition due to the lack of a described relationship.

### 2.2. MPOD Measurement

MPOD values were measured using MPS II ^®^ (Elektron Technology UK Ltd., Cambridge, UK).

This device utilizes the heterochromatic flicker photometry (HFP), which is a psychophysical technique that, as described by Murray et al. [12], involves a test stimulus that changes between two wavelengths: one is absorbed by macular carotenoids (blue at 460 nm) and the other is not absorbed (green at 540 nm). A flicker disk is shown, and the observer must press a button when the flicker is detected by the participant for a series of several blue-green ratios.

MPOD values range from 0 to 1. The device determines whether the task is successfully completed or not. If measures are reliable, it is highlighted in green; it is recommended to repeat in yellow, and measurements are not reliable in red. The MPOD task was carried out up to three times if the subject failed. After three fails, the participant was discarded.

### 2.3. Dietary Analysis

L and Z intake was evaluated using the KIDMED questionnaire, which is a test that allows for the evaluation of dietary habits in children and adolescents, developed by Serra-Majem et al. [11], and it is based on Mediterranean dietary patterns. The questionnaire has 16 dichotomous questions (YES/NO). Every KIDMED questionnaire was analyzed, obtaining a score according to the answers. The index ranged from 0 to 12 points, assigning −1 point to questions with a negative connotation and +1 point to questions with a positive connotation. Children were classified according to the Mediterranean diet adherence classification proposed by the KIDMED questionnaire (Table 1).

The KIDMED questionnaire was completed by the parents or legal guardians of the participants at home, and it was returned by the children at the time they were evaluated. Only fully completed questionnaires were considered. Incomplete questionnaires were discarded.

### 2.4. Statistical Analysis

Data analyses were performed using SPSS© Statistics software version 26.0 for Windows (IBM Corporation, Armonk, NY, USA).

A descriptive analysis was carried out, expressing the results as mean ± standard deviation. Data normality was assessed with the Kolmogorov–Smirnov test. As data did not follow a normally distribution, non-parametric tests were used. Differences between genders in all variables, as well as differences between answers, were evaluated with the Mann–Whitney U-test. The Kruskal–Wallis test was applied to determine differences between the Mediterranean diet adherence categories with Bonferroni’s post hoc adjustment. A correlation analysis between MPOD values and all variables was performed using Spearman’s Rho test.

For all tests, statistical significance was set at *p* < 0.05, establishing a 95% level of significance.

## 3. Results

Eighty-eight children (48 boys and 40 girls) were included in the current study. The participants’ measures are shown in Table 2. The mean age was 6.68 ± 0.70 years for the entire sample (range: 6–8 years). The mean MPOD value was 0.46 ± 0.18, although no statistically significant differences were found as a function of gender (*p* = 0.74).

**Figure 1:**
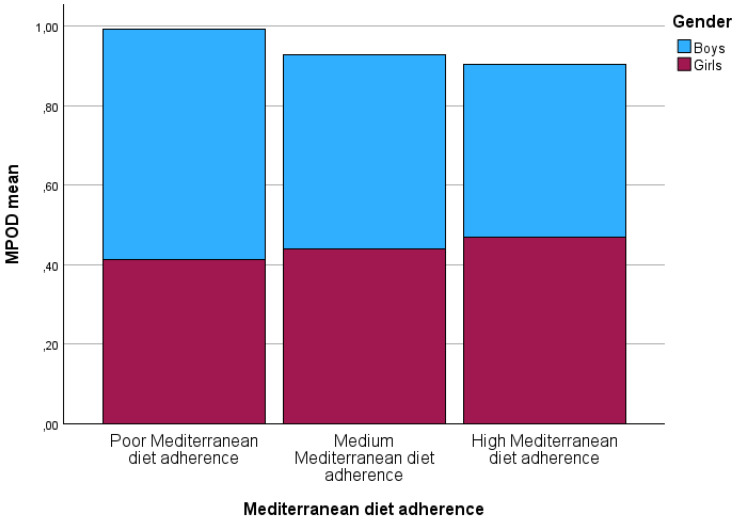
Macular pigment optical density values categorized according to Mediterranean diet ad-herence as a function of gender. MPOD: macular pigment optical density.

When the KIDMED questionnaires were analyzed, the mean score obtained was 7.19 ± 1.85 (range: between 3 points for minimum and 11 points for maximum); therefore, the average participant had a “medium” Mediterranean diet adherence. Nevertheless, the children were classified by their Mediterranean diet adherence (Figure 1). Only four children manifested “poor” Mediterranean diet adherence with a mean MPOD of 0.45 ± 0.95. Forty-two children showed medium and high Mediterranean diet adherence in each group, although when Kruskal–Wallis with Bonferroni’s post hoc adjustments were applied to determine differences among groups, no statistically significant differences were found in MPOD (*p* = 0.38), or between poor Mediterranean diet adherence and medium or high Mediterranean diet adherence (*p* = 0.84 and *p* = 0.86, respectively).

A Spearman’s Rho test was used to study correlations between variables (Table 3). No correlations were found for MPOD with several variables, such as Mediterranean diet adherence and KIDMED score.

On the other hand, the KIDMED questionnaire answers were evaluated question by question, obtaining MPOD values in each answer (YES/NO) (Table 4). The questions with a negative connotation (“Goes more than once a week to a fast-food (hamburger) restaurant” and “Skips breakfast”) showed that children answering YES had lower MPOD values than those who answered NO. The questions with a positive connotation (“Consumes fish regularly”; “Likes pulses and eats them more than once a week”; “Has cereals or grains for breakfast”; “Consumes nuts regularly”; “Has a dairy product for breakfast” and “Takes two yoghurts and/or some cheese daily”) had higher MPOD values when the participants answered YES. However, no significant differences were found in MPOD values between answers in any question (*p* > 0.05).

## 4. Discussion

The main purpose of the current study was to evaluate the relationship between the MPOD levels in macula lutea and the dietary intake of L and Z by adherence to a good diet pattern, such as the Mediterranean diet, which is rich in minerals, vitamins and xanthophyll carotenoids, being evaluated with a validated diet questionnaire focused on children.

Several studies have shown a positive correlation between L and Z consumption by dietary intake and MPOD levels, and even between L and Z serum and MPOD levels in adults [8,13,14,15,16]. This is an interesting finding to consider MPOD as a natural biomarker of L and Z in the human body, regarding retinal degenerative diseases due to oxidative processes and the improvement of cognitive function, as it is well known that a vast majority of L and Z accumulation is specifically deposited in brain areas such as the visual cortex and hippocampus, with different studies reporting a strong correlation between MPOD values and cognitive function [17].

However, in the child population, this relation remains unclear. Some studies have found a positive correlation between MPOD and the dietary intake of L and Z [18,19]. In contrast, most findings are in line with those of the present study, establishing no correlation between MPOD and the dietary intake of these carotenoids [10,20,21,22].

To our knowledge, Garcia-Romera et al. [10] were the first to use a novel tool (i.e., KIDMED questionnaire) to analyze and correlate food intake in childhood with the amount of macular carotenoids present in foods, which are deposited in the central retina, providing information about L and Z intake. Nevertheless, this association was evaluated in a larger sample in the present study compared to the previous study.

Most studies employed a three/seven-day food record [18,20,21,22]. However, we believe that this method is quite difficult to analyze due to the risk of bias and forgetting to adequately fill out the food record. This is the reason why we considered studying L and Z intake through this bi-answer questionnaire. On the other hand, Cannavale et al. [19] used the Block Food Frequency Questionnaire for children, which is a diet analyzer similar to ours, although we used the KIDMED questionnaire, since we considered it important to evaluate food intake based on adherence to the Mediterranean diet, which is rich in carotenoids (it is consumed chiefly in Spain and the rest of Southern Europe).

Surprisingly, when the differences among Mediterranean diet adherence categories were evaluated, no statistically significant differences were found. Moreover, when the KIDMED questionnaire responses were analyzed, some answers showed unexpected trends in their correlation with MPOD values. For instance, questions related to fruit or vegetable intake, such as “Takes a piece of fruit or fruit juice every day”, “Has a second piece of fruit every day” and “Has fresh or cooked vegetables regularly once a day”, showed higher MPOD values in the participants who answered NO, although no significant differences were found. This finding could be supported by the lack of correlation between MPOD and L and Z consumption [20,21,22] (in this case, with the KIDMED score and Mediterranean diet adherence). In addition, influential factors could play a key role in the adult population, but this may not be the case in the child population.

As was previously established, several risk factors are associated with lower MPOD, such as age, smoking, BMI and body fat percentage [23,24,25]. However, these risk factors could not apply, since children are not exposed to them yet. Due to the fact that no relationship was found between MPOD values and the dietary intake of food rich in L and Z in children, MPOD values may depend on genetic factors [26,27], mother’s dietary intake during pregnancy, and macular pigment deposition in the maternal uterus [28] or breastfeeding supply [29,30].

In addition, it is well known that adipose tissue competes with the retina for the uptake of L and Z [23,25]. This could also explain the lower MPOD measurements in the obese adult subjects. However, in the child population, there may bot be enough adipose tissue to compete or, perhaps, the pathways of L and Z differ from normality. Garcia-Romera et al. [10] also studied the effect of BMI on MPOD in children, although they did not find significant differences. However, it would be interesting to expand this study with a larger sample, in order to confirm this hypothesis.

Our study has several limitations. Firstly, BMI could be measured in order to compare overweight and healthy-weight children. In addition, it would have been interesting to study a larger age range or even to implement this study with teenagers, in order to determine the exact age at which it would be recommendable to intervene in L and Z intake.

## 5. Conclusions

In conclusion, the main finding of this study is that MPOD levels in children are not correlated with Mediterranean diet adherence and, thus, with good dietary habits. Genetic characteristics, mother’s dietary habits, oxidative stress and body fat composition in children could be the main factors influencing MPOD levels.

## Figures and Tables

**Table 1:** Mediterranean diet adherence classification according to the KIDMED questionnaire.

Mediterranean Diet Adherence Category	KIDMED Score
Poor Mediterranean diet adherence	≤3 points
Medium Mediterranean diet adherence	between 4 and 7 points
High Mediterranean diet adherence	≥8 points

**Table 2:** Participants’ characteristics.

	Total (n = 88)	Boys (n = 48)	Girls (n = 40)	*p*-Values
Age, years	6.68 ± 0.70	6.70 ± 0.68	6.65 ± 0.62	0.74
MPOD, d.u.	0.46 ± 0.18	0.46 ± 0.15	0.45 ± 0.19	0.47
KIDMED score	7.19 ± 1.85	7.15 ± 1.79	7.25 ± 1.93	0.72
Mediterranean diet adherence:				
Poor adherence	4	1	3	0.68
KIDMED score	3.00 ± 0.00	3.00 ± 0.00	3.00 ± 0.00	
MPOD value	0.45 ± 0.95	0.62 ± 0.00	0.41 ± 0.05	
Medium adherence	42	25	17	
KIDMED score	6.00 ± 0.88	5.88 ± 1.01	6.18 ± 0.63	
MPOD value	0.46 ± 0.15	0.47 ± 0.14	0.43 ± 0.16	
High adherence	42	22	20	
KIDMED score	8.79 ± 0.84	8.77 ± 0.75	8.80 ± 0.95	
MPOD value	0.45 ± 0.18	0.43 ± 0.15	0.47 ± 0.22	

Note: Data are presented as mean ± standard deviation (range). d.u.: density units; MPOD: macular pigment optical density.

**Table 3:** Spearman’s correlations among variables.

	Age	MPOD	KIDMED Score
Age	1.00	0.17 (0.10)	−0.14 (0.19)
MPOD	0.17 (0.10)	1.00	−0.09 (0.40)
KIDMED score	−0.14 (0.19)	−0.09 (0.40)	1.00

Note: Data are presented as Spearman’s ρ and *p* value. MPOD: macular pigment optical density.

**Table 4:** KIDMED questionnaire answers and macular pigment optical density value.

	MPOD Value	
Takes a piece of fruit or fruit juice every day	70 YES	0.44 ± 0.17
	18 NO	0.51 ± 0.13
Has a second piece of fruit every day	38 YES	0.44 ± 0.17
	50 NO	0.47 ± 0.16
Has fresh or cooked vegetables regularly once a day	56 YES	0.45 ± 0.17
	32 NO	0.47 ± 0.17
Has fresh or cooked vegetables more than once a day	20 YES	0.41 ± 0.17
	68 NO	0.47 ± 0.16
Consumes fish regularly (at least 2–3 times per week)	77 YES	0.46 ± 0.17
	11 NO	0.41 ± 0.14
Goes more than once a week to a fast-food (hamburger) restaurant	13 YES	0.45 ± 0.10
	75 NO	0.46 ± 0.17
Likes pulses and eats them more than once a week	83 YES	0.46 ± 0.17
	5 NO	0.41 ± 0.08
Consumes pasta or rice almost every day (5 or more times per week)	23 YES	0.42 ± 0.15
	65 NO	0.47 ± 0.17
Has cereals or grains (bread, etc.) for breakfast	73 YES	0.46 ± 0.16
	15 NO	0.45 ± 0.18
Consumes nuts regularly (at least 2–3 times per week)	18 YES	0.52 ± 0.21
	70 NO	0.44 ± 0.15
Uses olive oil at home	88 YES	0.46 ± 0.16
	0 NO	
Skips breakfast	2 YES	0.36 ± 0.02
	86 NO	0.46 ± 0.16
Has a dairy product for breakfast (yoghurt, milk, etc.)	79 YES	0.46 ± 0.16
	9 NO	0.43 ± 0.20
Has commercially baked goods or pastries for breakfast	26 YES	0.48 ± 0.14
	62 NO	0.44 ± 0.17
Takes two yoghurts and/or some cheese (40 g) daily	48 YES	0.46 ± 0.16
	40 NO	0.45 ± 0.17
Takes sweets and candy several times every day	2 YES	0.48 ± 0.00
	86 NO	0.45 ± 0.17

Note: Data are presented as mean ± standard deviation (range).

## Data Availability

Data are available upon request to corresponding author.
